# Subthalamic Nucleus High-Frequency Stimulation Restores Altered Electrophysiological Properties of Cortical Neurons in Parkinsonian Rat

**DOI:** 10.1371/journal.pone.0083608

**Published:** 2013-12-31

**Authors:** Bertrand Degos, Jean-Michel Deniau, Mario Chavez, Nicolas Maurice

**Affiliations:** 1 Team Dynamic and Pathophysiology of Neuronal Networks, Center for Interdisciplinary Research in Biology (CIRB), CNRS UMR7241/INSERM U1050, Collège de France, Paris, France; 2 Département de Neurologie – Centre de Référence Maladie de Parkinson, Hôpital Pitié-Salpêtrière, Assistance Publique – Hôpitaux de Paris (APHP), Paris, France; 3 Université Pierre et Marie Curie-Paris 6, CNRS-UMR-7225, Centre de Recherche de l'Institut du Cerveau et de la Moelle Epinière, Paris, France; McGill University, Canada

## Abstract

Electrophysiological recordings performed in parkinsonian patients and animal models have confirmed the occurrence of alterations in firing rate and pattern of basal ganglia neurons, but the outcome of these changes in thalamo-cortical networks remains unclear. Using rats rendered parkinsonian, we investigated, at a cellular level *in vivo*, the electrophysiological changes induced in the pyramidal cells of the motor cortex by the dopaminergic transmission interruption and further characterized the impact of high-frequency electrical stimulation of the subthalamic nucleus, a procedure alleviating parkinsonian symptoms. We provided evidence that a lesion restricted to the substantia nigra *pars compacta* resulted in a marked increase in the mean firing rate and bursting pattern of pyramidal neurons of the motor cortex. These alterations were underlain by changes of the electrical membranes properties of pyramidal cells including depolarized resting membrane potential and increased input resistance. The modifications induced by the dopaminergic loss were more pronounced in cortico-striatal than in cortico-subthalamic neurons. Furthermore, subthalamic nucleus high-frequency stimulation applied at parameters alleviating parkinsonian signs regularized the firing pattern of pyramidal cells and restored their electrical membrane properties.

## Introduction

Parkinson's disease (PD) motor symptoms including akinesia, rigidity and tremor result from the neurodegeneration of the nigro-striatal dopaminergic (DA) neurons. Based on anatomo-functional considerations, a pathophysiological model suggests that a loss of DA innervation causes an overactivity of the subthalamic nucleus (STN) glutamatergic neurons which project to the basal ganglia (BG) output structures, thus reinforcing the inhibitory influence they exert on the premotor thalamo-cortical network [1], [2]. Because STN lesion has beneficial motor effects in animal PD models and based on the idea that high-frequency stimulation (HFS) produces a functional inactivation of the stimulated area, it has been first proposed that HFS of the STN (STN HFS) improves parkinsonian motor functions by reducing STN overactivity thus re-activating the thalamo-cortical network [3]. Recent studies point against a reduction of STN and basal ganglia output structures activities with STN HFS, setting up the contrast of proposed mechanisms [4]–[7]. Despite general agreement that changes in the firing rate and dynamic properties of STN cells are central to PD motor symptoms, the repercussions of these changes in thalamo-cortical motor networks remain elusive.

To date, few studies aimed at recording the motor cortex activity at a cellular level in parkinsonian animal models, showing either no changes [8], [9], or a decrease [10]–[12] in the spontaneous single-unit firing rate. Experimental indirect measures exploring the functional consequences of DA loss on the cerebral cortex were mainly analyzed using global approaches such as functional cerebral imaging or electroencephalographic recordings [13]–[16]. These studies suggested major changes in spontaneous cortical activity but the observed effects call to reconsider the proposed cortical mechanisms underlying the motor impairments of PD and their restoration by STN HFS. Transcranial Magnetic Stimulation (TMS) and imaging studies in PD patients suggested that excitability of neurons in primary motor cortex was increased rather than decreased [17], [18]. Concerning the impact of STN HFS, functional cerebral imaging in PD patients during STN HFS revealed an overactivity of the thalamus as well as a reduction of primary motor and premotor cortices metabolic activity, an opposite effect to the expected result [19]–[23]. In line with this finding, a pharmacological blockade of substantia nigra *pars reticulata* (SNr) activity induces an increased discharge of thalamo-cortical neurons resulting in a decreased firing rate of motor cortex pyramidal cells [24] suggesting a major implication of cortical inhibitory interneurons in the cortical consequences of changes in BG activity. Thus, the net impact on the motor cortex of DA loss and STN HFS are still unclear.

We investigated at a cellular level the electrophysiological changes induced in the rat motor cortex by DA loss and by STN HFS. For this purpose, we combined *in vivo* single-cell extracellular and intracellular recordings to analyze the effects of substantia nigra *pars compacta* (SNc) lesion on membrane properties and firing of electrophysiologically identified pyramidal cells recorded in the orofacial motor cortex. The impact of STN HFS was determined by comparing, in SNc-lesioned rats, the activity and electrophysiological properties of pyramidal neurons recorded before and during application of the STN stimulation.

## Materials and Methods

### Ethic statements

All experiments were performed in accordance with local ethical committee (Institute of Biology, Center for Interdisciplinary Research in Biology and Collège de France; authorization #75–767) and EU Directive 2010/63/EU and every precaution was taken to minimize the stress, suffering and the number of animals used in each series of experiments. All animals used in this study were maintained on a 12:12-h light/dark cycle (lights on: 7:00 A.M. to 7:00 P.M.), with food and tap water available *ad libitum*.

At the end of each experiment, animals were sacrified using deep anesthesia with pentobarbital (130 mg/kg, i.p.; Ceva Santé Animale, Libourne, France).

### Unilaterally substantia nigra pars compacta lesioned animals

33 Sprague-Dawley rats weighing 150–175 g (Charles River Laboratories, L'Arbresle, France) were anesthetized with sodium pentobarbital (30 mg/kg i.p.) completed by injections of ketamine (27.5 mg/kg, i.m.; Imalgène, Mérial, Lyon, France) repeated as needed. Thirty minutes before the injection of 6-OHDA (6-hydroxydopamine, hydrochloride salt; Sigma), all animals received a bolus of desipramine dissolved in saline (25 mg/kg, i.p.; Sigma, Steinheim, Germany) to prevent neurotoxin-induced damage of noradrenergic neurons. Animals were fixed in a conventional stereotaxic head frame (Horsley-Clarke apparatus; Unimécanique, Epinay-sur-Seine, France). Wounds and pressure points were then infiltrated with lignocaine hydrochloride (Xylocaïne^®^ 2% inj., AstraZeneca, Monts, France). Body temperature was monitored by a rectal thermometer and maintained at 36.5°C with a homeothermic blanket (Harvard Apparatus, Kent, UK). A small craniotomy was made unilaterally (left side), and the overlying dura mater was removed. A single stereotaxic injection of 6-OHDA was delivered into the left substantia nigra *pars compacta* (SNc) [stereotaxic coordinates, anteriority from the interaural line (A): 3.7 mm, laterality from the midline (L): 2.1 mm, depth from the cortical surface (H): −7.55 mm], according to the stereotaxic atlas of Paxinos and Watson [25]. The neurotoxin 6-OHDA was dissolved immediately prior use in ice-cold 0.9% w/v NaCl solution containing 0.01% w/v ascorbic acid to a final concentration of 2.5 mg/ml. Then 4.0 µl of this 6-OHDA solution was injected at a rate of 16 µl/h through a steel canula (0.25 mm outside diameter) attached to a 10 µl Hamilton microsyringe (Cole-Parmer, London, UK) controlled by an electrical pump (KDS100; KD Scientific, Holliston, MA, USA). A delay of 5 minutes was observed between the time the canula was inserted in the SNc and the onset of the 6-OHDA injection. The canula was left in place ten minutes following the end of injection before removal. After surgery, rats received an intra-muscular injection of gentamicin to prevent bacterial infection (3 mg/kg, i.m.; Gentalline, Schering-Plough, Levallois-Perret, France).

### Animal preparation for electrophysiological recordings

During the third week after the 6-OHDA lesion, unilaterally-lesioned rats were anesthetized in an induction chamber containing isoflurane 4.5% (AErrane^®^, Baxter S.A., Lessines, Belgique). A canula was inserted into the trachea and isoflurane was delivered at 3–3.5%. Animals were thus placed in a stereotaxic frame. Wounds and pressure points were repeatedly (every 2 h) infiltrated with lignocaine (2%). All surgical procedures that consisted in drilling the bone above the orofacial motor cortex [A: 12.5 mm; L: 3.8 mm] and carefully removing the dura matter to gain access to the cortical area of interest were conducted under isoflurane anesthesia (Univentor 400, Univentor Limited, Zejtun, Malta). We performed the same anesthetic and surgical preparations for control animals (*n* = 27). For electrophysiological recordings, rats were subsequently maintained in a narcotized and sedated state by injections of fentanyl (4 µg/kg, i.p.; Janssen-Cilag, Issy-Les-Moulineaux, France) repeated every 20–30 min [26]–[30]. To obtain long-lasting stable intracellular and extracellular recordings, rats were immobilized with gallamine triethiodide (40 mg, i.m., every 2 h; Specia, Paris, France) and artificially ventilated (UMV-03, UNO, Zevenaar, The Netherlands). The degree of anesthesia was assessed by continuously monitoring the electrocorticogram (ECoG) and heart rate, and additional doses of fentanyl were administered at the slightest change toward a waking pattern (i.e., an increase in the frequency and reduction in amplitude of ECoG waves and/or an increase in the heart rate). Body temperature was maintained (36.5–37.5°C) with a homeothermic blanket. At the end of the experiments, animals received a lethal dose of sodium pentobarbital (150 mg/kg, i.p.).

### Electrophysiological recordings

In all cases, single-unit extracellular and intracellular recordings were performed while simultaneously recording the ECoG of the ipsilateral orofacial motor cortex. ECoG recordings were obtained with a low-impedance (≈ 60 kΩ) silver electrode placed on the dura above the orofacial motor cortex [A: 12.5 mm; L: 3.3–4 mm] [25], [31], [32]. The reference electrode was placed in the muscle on the opposite side of the head. Cortical cells, located in the orofacial motor cortex, were recorded within an area of 300 µm from the ECoG electrode [A: 12.5 mm; L: 3–4 mm].

Intracellular recordings were performed using glass micropipettes filled with 2 M potassium acetate (40–70 MΩ). For extracellular recordings, glass electrodes (10–20 MΩ) were filled with 0.5 M NaCl containing 1.5% of neurobiotin (Vector Laboratories, Burlingame, CA). Spontaneous activities of cortical neurons were recorded for at least 180 seconds before and during STN HFS. For each condition, we do not consider the first few seconds (at least 30 seconds) to ensure the stability of the cell for the periods of 150 seconds used for analysis. After each spontaneous activity, several measurements were performed in each condition for intracellularly recorded cells. Measurements of apparent membrane input resistance and time constant were based on the linear electrical cable theory applied to an idealized isopotential neuron [33]. The voltage-current (*V*-*I*) relationship was measured from variations of the membrane potential in response to intracellular injections of hyperpolarizing current pulses (−0.2 to −1.2 nA; 200 ms duration; every 1.0 s; *n* = 10) applied through the recording electrode. Apparent input resistance was measured from the mean (*n = *10) membrane potential change at the end of hyperpolarizing current pulses of low intensity (−0.4 nA; 200 ms duration; every 1.0 s) applied through the recording electrode or by measurement of the slope of the linear portion of the *V-I* curve. The values of membrane potential were corrected according to the potential recorded extracellularly immediately after termination of the intracellular recording. The membrane time constant, calculated from −0.4 nA current pulses, was the time taken for the membrane potential to reach 63% of its final value. The mean membrane potential was calculated from recording periods of 25 s.

We first used an antidromic cell identification to classify pyramidal cells as cortico-subthalamic (cortico-STN; *n* = 31) and cortico-striatal (*n* = 30) cells. We used the same characteristics of antidromic identification than in Degos et al. [34]. Briefly, antidromic spikes were characterized by their fixed latency at threshold, their collision with spontaneous discharges within appropriate time interval and their ability to follow HFS stimulation [35]. For 53 out of 114 recorded neurons, antidromic identification from both sites (subthalamus and striatum) failed probably because the axon or axon terminals of these neurons did not project exactly in the area of the stimulating electrode and therefore these neurons were classified as non-identified cells as in Li et al [12]. We also used a second *in vivo* classification based on the intrinsic firing patterns of the pyramidal cells in response to depolarizing current pulses [36], [37]. We classified neurons as regular spiking (RS), intrinsic bursting (IB) and non-inactivating bursting (NIB) cells [36], [37]. RS cells were characterized by a sustained discharge in response to depolarizing current pulses having an intensity >0.5 nA. IB cells presented successive spikes of decreasing amplitude and increasing duration, riding upon a slow depolarizing envelope. NIB cells presented, in response to depolarizing current pulses, all-or-none bursts of 3-8 action potentials that did not inactivate.

### Stimulation procedures

The right striatum [A: 8.7–9 mm; L: −(3.5–4.0) mm; H: 5.3 mm] and the left STN [A: 5.2 mm; L, 2.5 mm] respectively contralateral and ipsilateral to the recorded pyramidal cells were stimulated through bipolar coaxial stainless steel electrodes (diameter, 250 µm; tip-to-barrel distance, 300 µm; SNE-100; Rhodes Medical Instruments, Woodlands Hill, CA) positioned stereotaxically according to the atlas of Paxinos and Watson [25]. For antidromic stimulation, we stimulated at 1 Hz (see Fig. 9 in [34]). For electrical HFS, we used similar parameters than in Degos et al. [6] (130 Hz frequency; 60 µs width; 2–4 V (40–80 µA) intensity).

### Data acquisition

Extracellular and intracellular recordings were obtained using the active bridge mode of an Axoclamp 2B amplifier (Molecular Devices, Union City, CA). Data were sampled on-line on a computer connected to a CED 1401 interface using the Spike2 data acquisition program (Cambridge Electronic Design, Cambridge, UK) with a sampling rate of 25 kHz (intracellular signals), 10 kHz (extracellular signals), or 300 Hz (ECoG) for off-line analysis using Spike 2 (CED Software; Cambridge Electronic Design, Cambridge, UK).

As shown by Degos et al. (see Fig. 1 in [6]), spikes were not occulted by the stimulation artifacts because their duration was much longer than the period during which the amplifier was saturated. Briefly, during STN stimulation, spikes were firstly discriminated on-line from noise and stimulation artifacts on the basis of their amplitude, using the gate function (double threshold) of the discriminator (121 window discriminator; World Precision Instruments) and, secondly, all recordings were checked off-line to verify that all spikes were correctly sampled and that stimulation artifacts were not taken.

**Figure 1:**
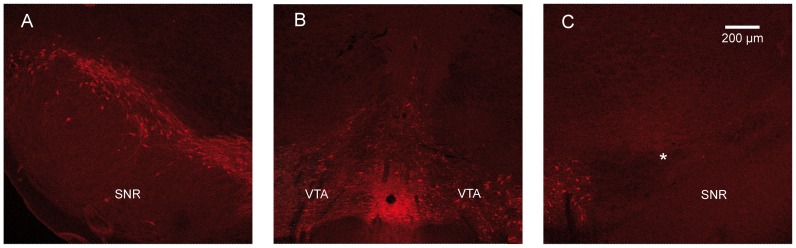
Histological controls of the 6-OHDA induced nigral denervation. Photomicrographs of TH-immunoreactivity at the SNc (***A*** and ***C***) and VTA (***B***) levels in unilaterally 6-OHDA lesioned rats. Note the sparing of the DA cell bodies in the VTA (***B***) and the complete loss of the DA cell bodies in the SNc ipsilateral to the lesion (***C***). Asterisks (*) indicate the loss of DA cell bodies in the 6-OHDA lesioned SNc. Scale bar in ***C*** (200 µm) applies in ***A***, ***B***. SNR: substantia nigra *pars reticulata*; VTA: ventral tegmental area.

### Data analysis

Interval interspike histograms of neuronal discharge were generated using the Spike2 software. The amplitude of action potentials was calculated as the potential difference between their voltage threshold, measured as the membrane potential at which the *dV*/*dt* exceeded 10 V.s^−1^ [38], and their peak. Numerical values are given as means ± SEM unless stated otherwise. Statistical significance was assessed by performing appropriate statistical tests (Student's *t* test completed if needed by Mann-Whitney Rank Sum test, a non parametric test). The normality of the membrane potential distribution was tested using the Kolmogorov-Smirnov test and a Gaussian-Laplace fit was performed. For statistical comparison between groups, the value of the “width” (“w”) of each Gaussian-Laplace fit was estimated. “w” corresponds to twice “sigma”, approximately 0.849 times the width of the peak at half height. Statistical analysis and curve fitting were performed using SigmaStat 3.0 (SPSS, Chicago, IL) and Origin 7.0 (Microcal Software, Northampton, MA), respectively.

### Discharge pattern analysis

Spontaneous activity of cortical neurons was analyzed by periods of 150 s. Epochs of elevated discharge rate were classified as bursts using a Poisson Surprise analysis [39], [40]. As described in Degos et al. [6], this was done using a script written for the Spike2 software. It is to note that, in the present study, cells are considered to be bursting when they present a transient increase of their spontaneous discharge activity. Thus, bursting cells evidenced by Poisson Surprise maximization do not necessarily correspond to the pyramidal cells classified as IB cells. This latter classification relies on electrophysiological criteria implicating activation of intrinsic conductance achieved by injecting depolarizing currents.

To quantify the degree of statistical association and the time delay between ECoG waves and intracellular activity of cortical neurons, we performed a statistical analysis of correlation. The nonlinear correlation coefficient h^2^ was calculated between these signals as function of a time shift (*τ*) [41], [42]. In contrast with classical methods, such as cross-correlation or coherence, which are only sensitive to linear relationships, this nonlinear index can reveal signal interdependencies under more general conditions [43]. To quantify the dependence of a signal *Y* on a signal *X*, an estimator of the nonlinear correlation index (*h* ^2^) was computed as follows:
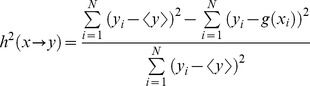
where *g*(*x_i_*) denotes a piecewise linear regression curve, used to predict the signal *Y* at any value of *X*, and 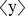 denotes the average of the time series *y_i_* over *N* samples. Statistically, this nonlinear correlation index quantifies the reduction of variance that can be obtained by predicting the *Y* values on the basis of the regression curve. The nonlinear index ranges between 0, when both signals are independent, and 1, for a perfect dependence. Theoretical and practical aspects of this method have been described in detail previously [41]. In the present study, the degree of association between ECoG and intracellular signals and the corresponding time delays during seizures were obtained by computing *h* ^2^ as a function of a time shift (*τ*) between the signals *y* and *x*. The shift for which the maximum of *h* ^2^(*τ*) was reached provided an estimate of the time delay between the activities [41], [43]. This analysis failed to demonstrate a coupling between ECoG rhythms and membrane variations.

### Histological assessment of 6-OHDA lesion

The extent of the 6-OHDA lesions was not tested prior the recordings by an apomorphine challenge because it is now well established that this procedure induces a priming phenomenon [44]. Instead, after completing the electrophysiological recordings, tyrosine hydroxylase (TH) immunohistochemistry was performed to assess the extent of the lesion. We previously described this method in details [16]. TH immunofluorescence was observed using a Leica LCS SP2 confocal microscope (Leica Microsystems, Wetzlar GmbH, Germany) equipped with 555-nm laser, differential interference contrast and Leica confocal software. Images (1024×1024) were acquired with a x10 Dry objective in sequential scanning mode. Only rats presenting a 6-OHDA lesion that was total in the SNc and the dorsal striatum but sparing the ventral tegmental area (VTA) and the nucleus accumbens (NAcc) were considered for further analysis.

## Results

### Histological control of the 6-OHDA-induced DA denervation

Histological control of the 6-OHDA induced lesion was performed in all rats (*n* = 30). As assessed by TH immunoreactivity (Fig. 1), the unilateral injection of 6-OHDA specifically in the SNc resulted in a complete loss of DA cell bodies located in the SNc but spared the VTA. Accordingly, DA terminals were lacking in the ipsilateral dorsal striatum but were preserved in the ipsilateral NAcc (data not shown). Only rats presenting such a circumscribed lesion were considered for further analysis. As expected, the lesion was specific of DA cell bodies and the GABAergic neurons from the adjacent SNR were spared as assessed by a Nissl staining (data not shown).

### Impact of SNc lesion on discharge characteristics of cortical neurons

To examine the consequences of DA loss on the firing rate and discharge pattern of cortical neurons, we performed *in vivo* extracellular single-unit and intracellular recordings in the orofacial motor cortex of rats bearing a unilateral 6-OHDA lesion of the SNc. Firing rates, discharge patterns as well as active and passive membrane properties were analyzed and compared with those obtained in control rats. Discharge pattern characteristics were analyzed using the Poisson Surprise maximization that provides, using the mean discharge rate as a criterion, the temporal occurrence of bursts and global descriptive indices about the neuronal discharge (see materials and methods). Because in both control and SNc-lesioned conditions, we did not find statistical differences in the discharge frequency and discharge pattern between the neurons recorded using extracellular or intracellular recordings (Table 1), we pooled all data together to determine the discharge characteristics. Moreover, using intracellular recordings, each recorded cell in both control (*n* = 23) and SNc-lesioned animals (*n* = 27) was classified as regular spiking (RS), non-inactivating bursting (NIB) or intrinsic bursting (IB) according to previously *in vivo* defined criteria [36], [37]. The proportion of each category was not significantly modified following the 6-OHDA lesion of the SNc (RS: 65.3% vs. 70.3%; NIB: 21.7% vs. 22.2%; IB: 13.0% vs. 7.5% in control and lesioned animals, respectively; Chi-square, *p = *0.801).

**Table 1:** Discharge rate and pattern of cortical cells recorded using single-unit extracellular or intracellular recordings in control and SNc-lesioned animals were similar.

		N	Mean discharge frequency (Hz)	Percentage of spikes emitted during bursts	Mean bursts occurrence (bursts/min)	Mean intraburst frequency (Hz)
**Control condition**	Extracellular recordings	29	1.6±0.3	66.6±2.3	8.9±1.4	9.2±1.2
	Intracellular recordings	23	1.9±0.5^(1)^	64.8±3.2^(2)^	9.8±2.3^(3)^	10.1±3.1^(4)^
**SNc-lesioned condition**	Extracellular recordings	35	4.6±0.6	61.4±2.4	22.4±3.0	20.4±3.1
	Intracellular recordings	27	4.9±0.8^(5)^	58.5±3.5^(6)^	18.7±3.5^(7)^	15.5±2.6^(8)^

No statistical differences were found between extracellular and intracellular recordings; *t-test*, ^(1)^ *p* = 0.614; ^(2)^ *p* = 0.640; ^(3)^ *p* = 0.752; ^(4)^ *p* = 0.774; ^(5)^ *p* = 0.761; ^(6)^ *p* = 0.493; ^(7)^ *p* = 0.433; ^(8)^ *p* = 0.253.

**Figure 2:**
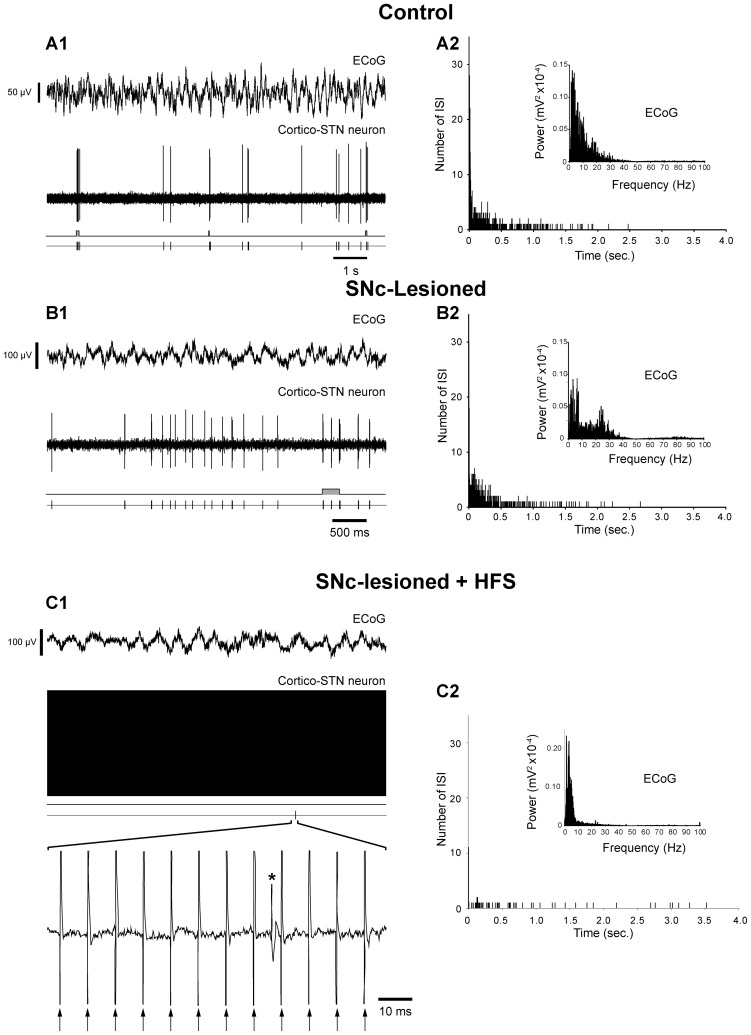
Effect of SNc lesion and impact of STN HFS on the spontaneous activity of cortico-STN cells and on the electrocorticogram (ECoG) of the orofacial motor cortex. The spontaneous activity of a cortico-STN neuron was extracellularly recorded simultaneously with the ECoG signal of the orofacial cortex in control rats (***A***), and in SNc-lesioned animals, before (***B***) and during STN HFS (***C***). Note that the SNc lesion induces an increased firing rate of the cortico-STN neuron (***B1***) that is accompanied by an increased number of bursts and by the appearance of an excessive synchronization in beta frequency band in the ECoG (***B2, inset***) compared to control situation (***A1, A2, inset***). These SNc lesions-induced changes were abolished during STN HFS (***C***). In panels ***A1*** **,** ***B1***, and ***C1***, the different traces correspond, from the top to the bottom, to the ECoG signal, the simultaneous extracellular recording of the cortico-STN cell, the bursting discharge detected by Poisson Surprise analysis (*S*≤2) and the discharge of the cell represented as a sequence of spikes. In panels ***A2*** **,** ***B2***, and ***C2***, histograms display the corresponding interspike intervals and insets show the power spectrum (FFT) of the corresponding ECoGs. In ***C1***, arrows indicate the stimulation artifacts and the asterisk an action potential. Abbreviation: ECoG: electrocorticogram; FFT: Fast Fourier Transform; HFS: high-frequency stimulation; ISI: interspike interval; SNc: substantia nigra pars compacta; STN: subthalamic nucleus.

**Figure 3:**
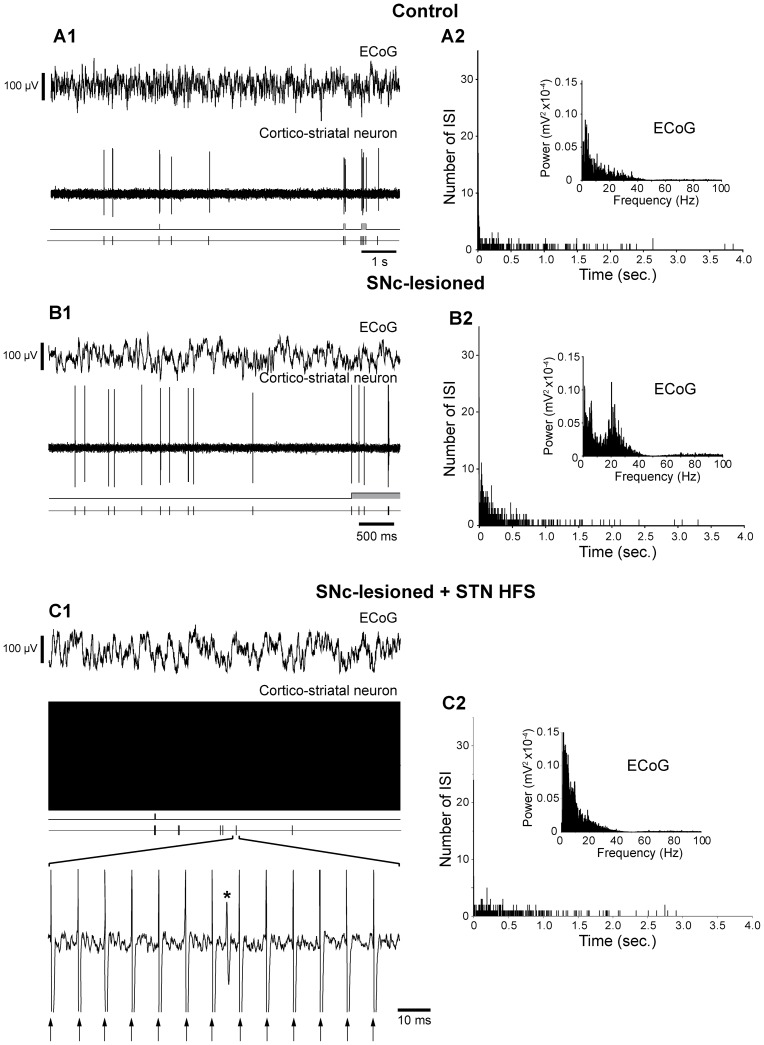
Effect of SNc lesion and impact of STN HFS on the spontaneous activity of cortico-striatal cells and the electrocorticogram (ECoG) of the orofacial motor cortex. The spontaneous activity of a cortico-striatal neuron was extracellularly recorded simultaneously with the ECoG signal of the orofacial cortex in control rats (***A***), and in SNc-lesioned animals, before (***B***) and during STN HFS (***C***). Note that the SNc lesion induced an increased firing rate of the cortico-striatal neuron (***B1***) that was accompanied by an increased number of bursts and by the appearance of an excessive hypersynchronisation in the beta frequency band in the ECoG (***B2, inset***) compared to control situation (***A1, A2, inset***). These SNc lesions-induced changes were abolished during STN HFS (***C***). In panels ***A1*** **,** ***B1***, and ***C1***, the different traces correspond, from the top to the bottom, to the ECoG signal, the simultaneous extracellular recording of the cortico-striatal cell, the bursting discharge detected by Poisson Surprise analysis (*S*≤2) and the discharge of the cell represented as a sequence of spikes. In panels ***A2*** **,** ***B2***, and ***C2***, histograms display the corresponding interspike intervals and insets show the power spectrum (FFT) of the corresponding ECoGs. In ***C1***, arrows indicate the stimulation artifacts and the asterisk an action potential. Abbreviation: ECoG: electrocorticogram; FFT: Fast Fourier Transform; HFS: high-frequency stimulation; ISI: interspike interval; SNc: substantia nigra pars compacta; STN: subthalamic nucleus.

**Figure 4:**
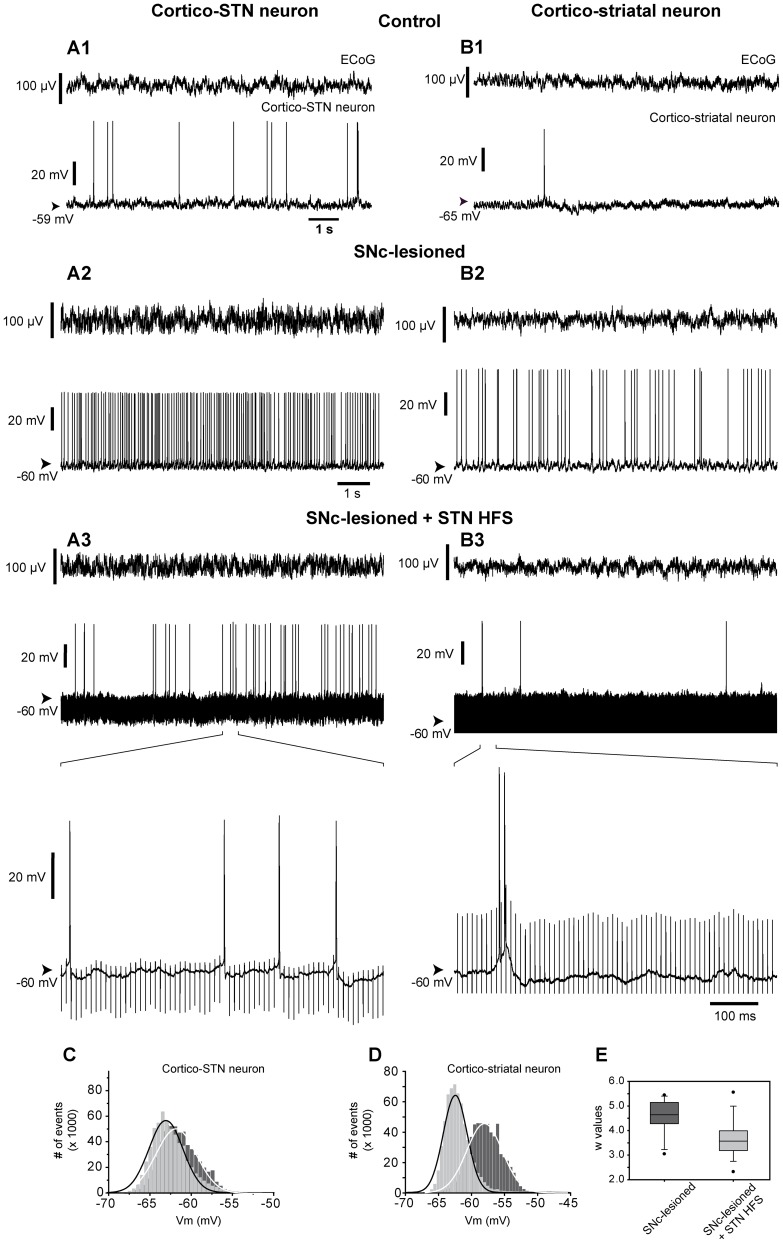
Intracellularly recorded spontaneous activity of identified pyramidal cells and simultaneously recorded ECoG in the orofacial motor cortex in intact and SNc-lesioned rats, before and during STN HFS. Activity of cortico-STN (***A***) and cortico-striatal (***B***) neurons were recorded simultaneously with the ECoG from the orofacial motor cortex of intact rats (***A1*** **,** ***B1***) and of SNc-lesioned animals, before (***A2*** **,** ***B2***) and during (***A3*** **,** ***B3***) STN HFS. In each panel ***A1*** **,** ***A2*** **,** ***A3*** **,** ***B1*** **,** ***B2*** and ***B3***, the top trace is the ECoG signal and the bottom trace is the simultaneous intracellular recording of the pyramidal cell. Note that changes of firing rate and pattern were similar to those observed using extracellular recordings. In ***A1*** **,** ***A2*** **,** ***A3*** **,** ***B1*** **,** ***B2*** and ***B3***, the values of the membrane potential are indicated on the left. Note that SNc lesion resulted in a more depolarized membrane potential. (***C*** **,** ***D***) Example of the impact of STN HFS on the distribution of the membrane potential of the cortico-STN (***C***) and the cortico-striatal (***D***) neurons recorded in SNc lesioned animals and illustrated in (***A2, A3***) and (***B2, B3***), respectively. In lesioned rats (black bars), the membrane potential of this cortico-STN neuron was unimodally distributed around a mean value of −61.8 mV that was best fitted by a Gaussian function (*r* ^2^  = 0.98; *n* = 6.25×10^5^ values; bin size: 0.5 mV) and shifted to more hyperpolarized values during STN HFS (light gray bars) (mean, −64.0 mV; *n* = 6.25×10^5^ values; bin size, 0.5 mV; Gaussian fit, *r* ^2^  = 0.95). In lesioned rats (black bars), the membrane potential of this cortico-striatal neuron was unimodally distributed around a mean value of −58.0 mV that was best fitted by a Gaussian function (*r* ^2^  = 0.99; *n* = 6.25×10^5^ values; bin size: 0.5 mV) and shifted to more hyperpolarized values during STN HFS (light gray bars) (mean, −62.5 mV; *n* = 6.25×10^5^ values; bin size, 0.5 mV; Gaussian fit, *r* ^2^  = 0.97). (***E***) Box plot summary of the impact of STN HFS on the value “w” that was estimated for each Gaussian-Laplace fit of the distribution of the membrane potentials in all pyramidal neurons (see materials and methods) indicating a narrowing of the distribution of membrane potential during STN HFS. Abbreviation: ECoG: electrocorticogram; HFS: high-frequency stimulation; SNc: substantia nigra pars compacta; STN: subthalamic nucleus.

In control animals, the mean firing frequency of pyramidal cells was 1.7±0.3 Hz [*n* = 52 among which 29 were recorded extracellularly (Fig. 2A and 3A) and 23 intracellularly (Fig. 4A1 and 4B1)]. As evidenced by Poisson Surprise analysis, among these 52 cells, only 4 presented a regular discharge whereas 48 cells exhibited some bursts whose characteristics are detailed in Table 1. Following SNc lesion, the discharge frequency of the ipsilateral cortical cells was significantly increased with a mean discharge of 4.7±0.5 Hz [*n* = 62 among which 35 were recorded extracellularly (Fig. 2B and 3B) and 27 intracellularly (Fig. 4A2 and 4B2); *p*<0.001; Mann-Whitney Rank Sum Test; Table 1]. Except for the one cell that was regularly discharging, there was a marked increase of the mean bursts occurrence [9.1±1.2 bursts/min in control, *n* = 1059 bursts recorded in 48 cells and 20.5±2.3 bursts/min after lesion, *n* = 3149 bursts analyzed in 61 cells recorded in SNc-lesioned rats; *p*<0.001; Mann-Whitney Rank Sum Test; Table 1]. Furthermore, the mean duration of the bursts was significantly decreased (0.9±0.10 seconds in control versus 0.5±0.02 seconds after lesion; *p*<0.001, Mann-Whitney Rank Sum test) and the mean intraburst frequency was significantly increased compared to control (9.6±1.4 Hz in control and 18.3±2.1 Hz after lesion; *p*<0.001; Mann-Whitney Rank Sum Test).

**Figure 5:**
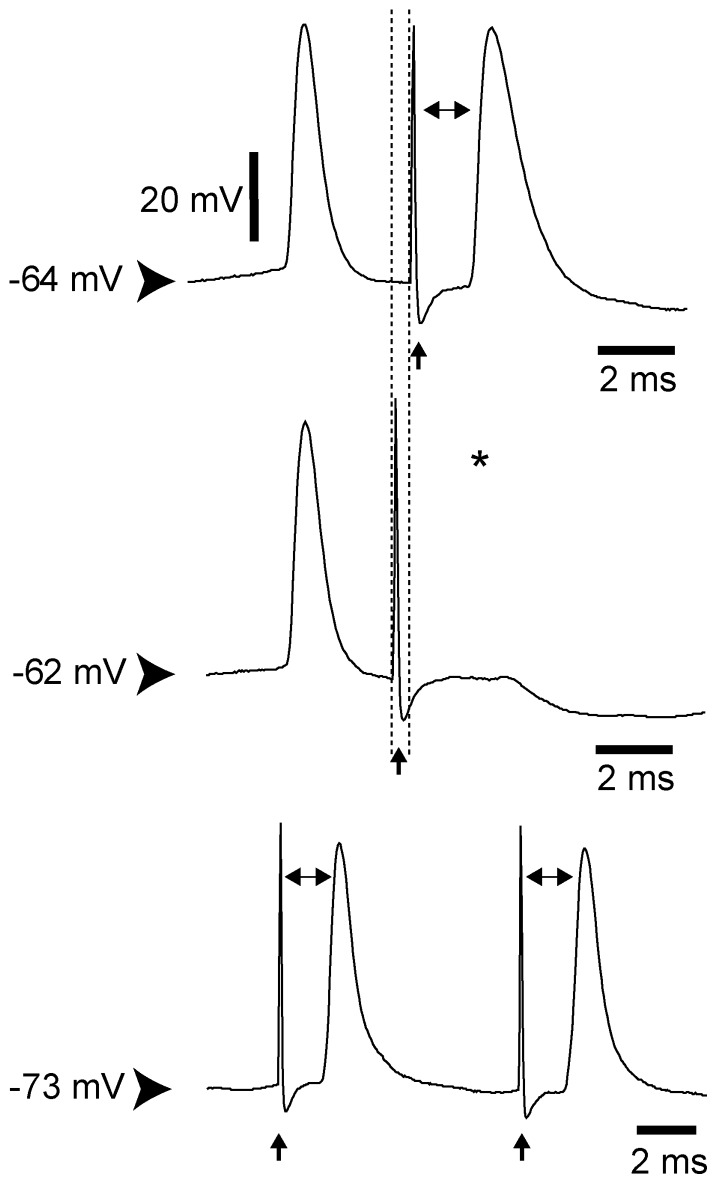
Electrophysiological identifications of pyramidal neurons. Cortico-STN and cortico-striatal neurons were identified by their antidromic activation following electrical stimulation of the ipsilateral STN and contralateral striatum, respectively. Intracellular characterization of the antidromic spike evoked in a pyramidal cell by ipsilateral STN stimulation: The antidromic latency (1.5 ms), which was measured as indicated by the double-headed arrows, was not affected by DC-induced hyperpolarization (from −64 to −73 mV; *i*_DC_ −0.5 nA). The top trace shows no collision between orthodromic and antidromic spikes when the STN stimulation is applied 3.3 ms after a spontaneous orthodromic action potential. The middle trace shows a collision of an antidromic spike (asterisk) with a spontaneous orthodromic action potential occurring at an appropriate time interval. In each trace, the values of the membrane potential are indicated on the left.

To specifically address the respective impact of SNc lesion on the activity of cortico-STN and cortico-striatal neurons, the pyramidal cells were identified as projecting either to the STN or to the striatum using the antidromic activation method (Fig. 5). Firing characteristics of these two cell populations are detailed in Table 2. In control rats, no significant difference in firing rate was found between cortico-STN and cortico-striatal neurons. Following SNc lesion and compared to control condition cortico-STN and cortico-striatal neurons showed an increase in mean firing rates, mean bursts occurrences and mean intraburst frequencies, associated with decreased mean burst durations (for more details see Table 2).

**Table 2:** Impact of SNc lesions on the discharge rate and pattern of cortico-STN and cortico-striatal cells.

	Cortico-subthalamic cells		Cortico-striatal cells	
	Control	SNc-lesioned	Control	SNc-lesioned
**Antidromic latency (ms) (** ***Range ; n*** **)**	2.1±0.2 (*1.4–4.0; 11*)	1.9±0.1 (*0.9–3.2; 20*)	7.7±0.5 (*5.0–10.4; 13*)	6.1±0.5 (*2.6–9.2; 17*)
**Mean discharge frequency (Hz)**	2.6±0.7	4.3±0.7(1)	1.3±0.4	4.9±1.0*(2)
**Nb of bursting cells/total cells (Total nb of bursts)**	11/11 (258 bursts)	20/20 (990 bursts)	12/13 (203 bursts)	16/17 (938 bursts)
**Mean bursts occurrence (bursts/min.)**	9.5±2.3	19.8±3.4*(3)	6.9±1.9	23.5±4.9*(4)
**Mean burst duration (sec.)**	0.9±0.1	0.5±0.1*(5)	0.8±0.1	0.4±0.03(6)
**Mean intraburst frequency (Hz)**	11.8±2.6	15.6±3.0(7)	8.0±1.7	22.2±4.7*(8)

Ms: millisecond; Hz: Hertz; nb: number; sec: second; min: minute; *: significant result.

^(1)^ : no difference (*p* = 0.107); t-test.

^(2)^ : *p* = 0.012; Mann-Whitney Rank Sum test.

^(3)^ : *p* = 0.044; t-test.

^(4)^ : *p* = 0.007; Mann-Whitney Rank Sum test.

^(5)^ : *p*<0.001; Mann-Whitney Rank Sum test.

^(6)^ : no difference (*p* = 0.304); Mann-Whitney Rank Sum test.

^(7)^ : no difference (*p* = 0.408); t-test.

^(8)^ : *p* = 0.019; Mann-Whitney Rank Sum test.

### Impact of STN HFS on the firing rate and pattern of cortical neurons in SNc-lesioned rats

To investigate the impact of STN HFS on the alterations induced in pyramidal cells by DA loss, single-unit recordings were performed in the orofacial motor cortex of SNc-lesioned rats before and during STN HFS (Fig. 2C, 3C, 4A3 and 4B3). For comparison purpose, only data obtained in cells successively recorded in these two conditions were retained to further analysis. The parameters of STN stimulation (2–4 V, 60 µs, 130 Hz) were chosen on the basis of the motor improvement obtained in rats submitted to pharmacological blockade of DA transmission (neuroleptics) or SNc lesion (6-OHDA). In behaving animals, these parameters allow to relieve the catalepsy induced by neuroleptics [6] as well as the akinesia of the forelimb contralateral to a unilateral SNc lesion [45].

STN HFS had a dramatic impact on the firing rate of the cortical neurons. Indeed, the mean firing rate of the pyramidal cells decreased significantly from 4.7±1.1 Hz (*n* = 14 intracellularly recorded cells) before STN HFS to 1.1±0.3 Hz (*n* = 14) during the stimulation (*p = *0.002; Mann-Whitney Rank Sum test). To further characterize the impact of STN HFS on the cortical cells, their pattern of discharge was analyzed using the Poisson Surprise maximization. Most of the pyramidal cells intracellularly recorded before and during STN HFS in SNc-lesioned rats (Fig. 4) displayed some bursts (13 out of 14). STN HFS markedly altered the firing pattern of these cortical cells. First, STN HFS clearly decreased the number of bursts. Among the 13 cells that presented some detectable bursts before STN stimulation, 3 became regularly firing during STN HFS and in the 10 cells still bursting, the number of bursts strongly decreased (from 15.2±3.4 bursts/min before stimulation to 3.7±1.6 bursts/min during the STN stimulation; *n* = 13; *p = *0.003, Mann-Whitney Rank Sum test). Furthermore, STN HFS reversed significantly the effect of DA lesion on mean burst duration (from 0.6±0.1 second prior stimulation to 1.4±0.3 second during STN HFS; *p = *0.008, Mann-Whitney Rank Sum test) and on mean intraburst frequency (from 65.8±3.6 Hz, *n* = 533 bursts from 13 cells before stimulation to 59.0±8.8 Hz, *n* = 130 bursts from 10 cells during STN HFS; *p*<0.001, Mann-Whitney Rank Sum test).

We then test whether STN HFS differently impacted the two subpopulations of pyramidal cells. Among the 14 intracellularly recorded cortical cells, 6 were identified as projecting to the STN (Fig. 4A), 5 as innervating the striatum (Fig. 4B) and 3 could not be antidromically driven from either structure. STN HFS differentially modified the firing rate and pattern of discharge of these two populations of cortical cells (Table 3). Indeed, during STN HFS, the mean discharge rate of cortico-striatal neurons was significantly reduced (Fig. 4B3) whereas the mean discharge rate of cortico-STN neurons was not significantly altered (Fig. 4A3). Concerning their discharge pattern, cortico-STN neurons presented less bursts during STN HFS, with significant increased mean burst duration and decreased mean intraburst frequency. Except an unaffected mean intraburst frequency, cortico-striatal neurons presented similar phenomenon as cortico-STN neurons.

**Table 3:** Impact of STN HFS on the discharge rate and pattern of cortico-STN and cortico-striatal cells recorded in SNc-lesioned rats.

	Cortico-subthalamic cells		Cortico-striatal cells	
	SNc-lesioned	SNc-lesioned + STN HFS	SNc-lesioned	SNc-lesioned + STN HFS
**Antidromic latency (ms) (** ***Range; n*** **)**	1.3±0.1 (*0.9–1.5; 6*)		7.2±1.0 (*4.2–9.2; 5*)	
**Mean discharge frequency (Hz)**	4.9±1.8	0.9±0.6(1)	6.1±2.0	0.1±0.1^*(2)^
**Nb of bursting cells/total cells (Total nb of bursts)**	6/6 (193 bursts)	4/6 (48 bursts)	5/5 (266 bursts)	4/5 (10 bursts)
**Mean bursts occurrence (bursts/min.)**	12.9±3.8	3.2±1.3*(3)	26.6±7.4	1.0±0.4*(3)
**Mean burst duration (sec.)**	0.7±0.02	1.7±0.1*(3)	0.4±0.03	5.4±2.8*(3)
**Mean intraburst frequency (Hz)**	13.3±3.2	4.2±1.4*(4)	18.9±7.9	6.4±5.7(5)

Ms: millisecond; Hz: Hertz; nb: number; sec: second; *: significant result.

^(1)^ : no difference (*p* = 0.059); t-test.

^(2)^ : *p* = 0.041; Paired t-test.

^(3)^ : *p*<0.001; Mann-Whitney Rank Sum Test.

^(4)^ : *p* = 0.026; t-test.

^(5)^ : no difference (*p* = 0.31); Mann-Whitney Rank Sum Test.

**Figure 6:**
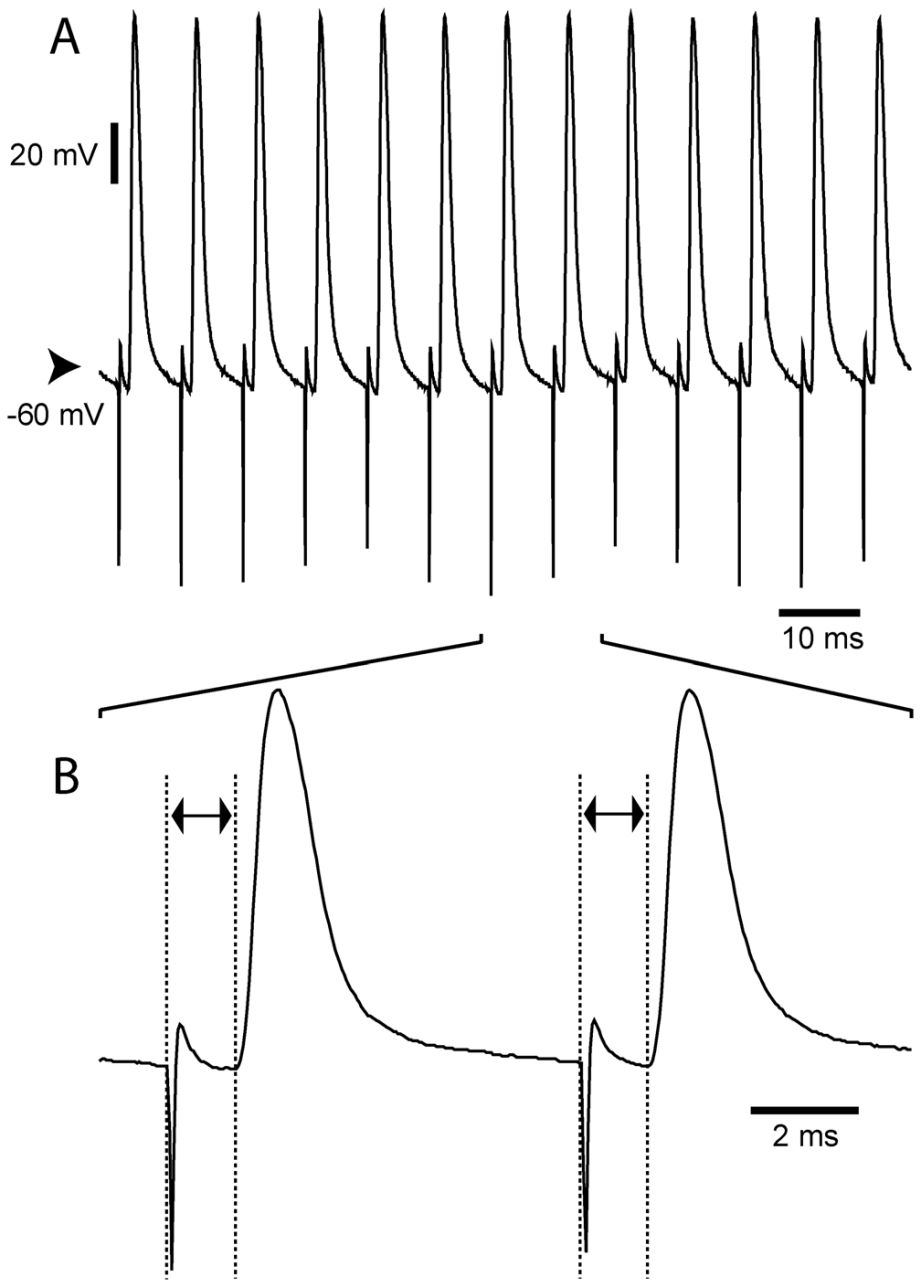
Long lasting antidromic activation of cortico-subthalamic neurons during STN HFS. (***A***) Recording of one cortico-STN neuron during STN HFS indicating that this cell was reliably antidromically driven from the STN during the entire period of the stimulation. Note that the antidromic spikes were evoked independently of the cell membrane potential. (***B***) Magnified view of the recording illustrating the fixed latency of the antidromic spikes (latency  = 1.3 ms) as marked by the double-headed arrows.

Interestingly, some cortico-STN neurons were antidromically activated by STN stimulation at very low threshold intensity. Figure 6 illustrated the case of one cortico-STN neuron from which an intensity of 4 V reliably drove an antidromic activation, the antidromic spike being present after each STN stimulation with a fixed latency. To avoid this 130 Hz antidromic activation for analysis, we reduced at 2 V the intensity of STN stimulation. Therefore, as already described for a subset of cortico-STN neurons by Li et al. [12], [46], each stimulation pulse delivered in the STN could potentially evoke an antidromic spike in the cortical neuron. As a result, during the STN HFS, these neurons displayed a regular discharge, imposed by the antidromic activation, at the frequency of STN stimulation. However, during the train of stimulations, some stimuli could fail to fire antidromically the soma but only transiently. This might be due either to a collision phenomenon between an orthodromic spike and the evoked antidromic spike, or to an axonal failure [47].

### Impact of SNc lesion on the electrical membrane properties of pyramidal neurons

**Figure 7:**
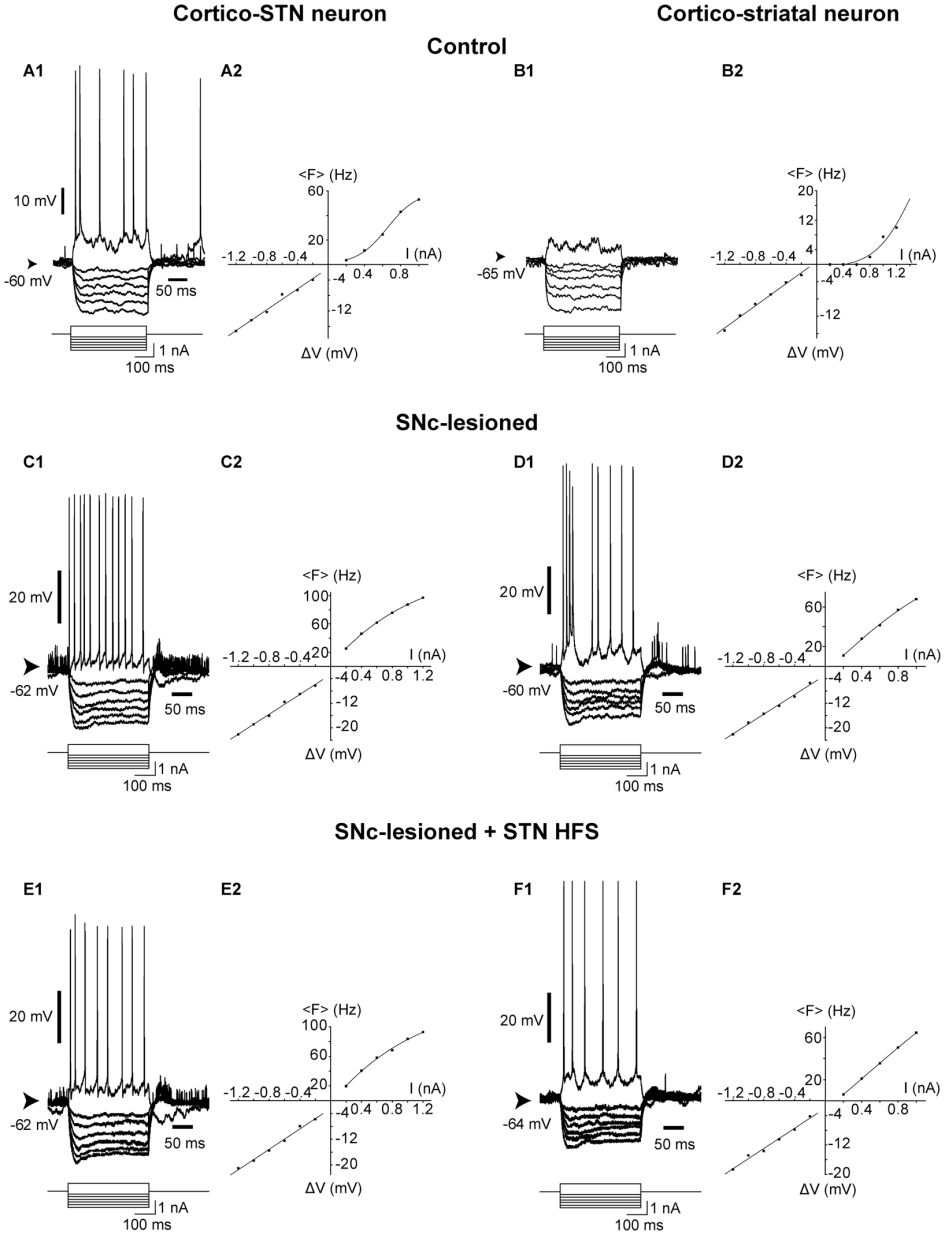
Impact of SNc lesion on electrical membrane properties of cortico-subthalamic (*A, C* and *E*) and cortico-striatal (*B, D* and *F*) neurons in control and SNc-lesioned situations, before and during STN HFS. *A1*, *B1*, *C1* and ***D1***, Voltage responses of pyramidal neurons (top traces) to intracellular injection of positive and negative square current pulses (bottom traces) in control (***A1*** **,** ***B1***) and SNc-lesioned (***C1*** **,** ***D1***) situations. The responses to the negative current pulses are an average of 10 successive trials. ***A2*** **,** ***B2*** **,** ***C2*** and ***D2***, Plots of the voltage changes (Δ*V*) (bottom) and of the mean firing frequency (<*F>*) (top) as a function of current intensity in control (***A2*** **,** ***B2***) and SNc-lesioned (***C2*** **,** ***D2***) rats. ***E*** and ***F*** are the voltage responses and the corresponding plots of the voltage changes and mean firing frequency as a function of current intensity obtained in the same cells shown in C and D, respectively, but during STN HFS. The apparent input resistance was measured from the linear portion of the *V*-*I* curve. The *F*-*I* relationship was best fitted by a sigmoidal function (*r^2^* >0.99). Abbreviation: HFS: high-frequency stimulation; STN: subthalamic nucleus; SNc: substantia nigra *pars compacta.*

As reported above, the degeneration of dopaminergic neurons of the SNc generated an increase in the firing frequency and burst discharges of pyramidal cells of the motor cortex, changes which were overcome by the STN HFS. To further define the changes of neuronal excitability underlying these effects, passive and active electrical membrane properties of pyramidal neurons in control (*n* = 23 cells from 14 rats), SNc-lesioned rats (*n* = 27 cells from 21 rats) and SNc-lesioned rats submitted to STN HFS (*n* = 14 cells from 10 rats) were compared using *in vivo* intracellular recordings (Fig. 7).

In control rats, the recorded population of pyramidal cells (Table 4) presented a membrane potential (−62.8±0.9 mV) whose distribution was best fitted by a Gaussian function exhibiting a mean “w” value of 4.1±0.2 (see Material and Methods). The V-I relationship, which was determined by measuring membrane potential changes in response to a series of intracellular square current pulses (Fig. 7) was then examined. The apparent input resistance, measured from the linear portion of the V-I plot was 16.1±2.1 MΩ in intact rats. The SNc lesion had a marked impact on some of the electrical membrane properties of pyramidal cells (for more details see Table 4). Indeed, in SNc-lesioned animals, the membrane potential was significantly more depolarized (−57.8±0.7 mV) than in control situation. SNc lesions significantly modified the threshold and the duration of action potentials but not others membrane properties. However, compared to the control condition, the amplitude of membrane potential fluctuations of the pyramidal cells recorded in SNc-lesioned animals was statistically increased as indicated by the significant increase of “w” (4.7±0.2; *n* = 27; *p = *0.019, t-test).

**Table 4:** Comparison of membrane properties of pyramidal cells in control and SNc-lesioned rats.

	Vm (mV)	Rin (MΩ)	τ (ms)	AP threshold (mV)	AP amplitude (mV)	AP duration (ms)
**Control (** ***n*** **)**	−62.8±0.9 (*23*)	16.1±2.1 (*8*)	9.0±0.8 (*9*)	−54.6±1.3 (*20*)	60.6±2.4 (*20*)	2.0±0.1 (*20*)
**SNc-lesioned (** ***n*** **)**	−57.8±0.7(1)(*27*)	17.1±0.6 (*18*)	8.8±0.7 (*19*)	−50.5±0.8(2) (*26*)	60.8±1.4 (*26*)	1.7±0.1(3)(*26*)

AP: Action potential; mV: millivolt; Rin: membrane input resistance; MΩ: megohm; τ: membrane time constant; ms: millisecond; Vm: mean membrane potential.

^(1)^ : *p*<0.01; t-test.

^(2)^ : *p* = 0.008; t-test.

^(3)^ : *p* = 0.003; t-test.

As detailed in Table 5, among the 23 intracellularly recorded neurons in intact rats, 5 were antidromically identified as projecting to the STN (Fig. 7A) and 4 as innervating the striatum (Fig. 7B). Among the 27 intracellularly recorded pyramidal cells in SNc-lesioned rats, 7 were identified as projecting to the STN (Fig. 7C) and 8 as innervating the striatum (Fig. 7D). Interestingly, SNc lesions differently affected the membrane properties of the two cell populations. Whereas all the membrane properties measured in cortico-STN neurons were unchanged following SNc lesion (Fig. 7C), the lesion increased the discharge rate and the membrane input resistance of cortico-striatal neurons, and induced a depolarization of their membrane potential (Fig. 7D and Table 5).

**Table 5:** Comparison of membrane properties of cortico-STN and cortico-STR cells in control and SNc-lesioned rats.

	Cortico-subthalamic cells		Cortico-striatal cells	
	Control	SNc-lesioned	Control	SNc-lesioned
**Antidromic latency (ms) (** ***Range; n*** **)**	1.7±0.1 (*1.4–2.0; 5*)	1.2±0.1 (*0.9–1.5; 7*)	7.3±0.9 (*5.9–9.9; 4*)	6.2±0.7 (*4.2–9.2; 8*)
**Vm (mV)**	−60.9±2.5	−58.1±1.5	−65.3±2.4	−57.2±1.2(1)
**Rin (MΩ)**	16.0±2.3	14.7±1.1	10. 3±2.5	17.2±1.2(2)
**τ (ms)**	7.6±0.7	8.7±1.5	6.1±1.4	8.5±1.0
**AP threshold (mV)**	−53.6±2.2	−51.5±2.0	−55.1±2.6	−50.6±1.0
**AP amplitude (mV)**	65.4±2.4	58.8±3.5	53.7±4.1	63.5±2.7
**AP duration (ms)**	1.8±0.2	1.7±0.1	2.2±0.1	1.7±0.1(3)

AP: Action potential; mV: millivolt; Rin: membrane input resistance; MΩ: megohm; τ: membrane time constant; ms: millisecond; Vm: mean membrane potential.

^(1)^ : *p* = 0.007; t-test.

^(2)^ : *p* = 0.029; t-test.

^(3)^ : *p* = 0.016; t-test.

### Impact of STN HFS on membrane properties of pyramidal neurons in SNc-lesioned rats

As reported above, STN HFS had a marked inhibitory influence on the discharge rate of pyramidal cells. To test whether this inhibitory influence might result from alterations of the intrinsic excitability of pyramidal cells due to STN HFS, passive and active membrane properties of pyramidal cells in SNc-lesioned rats were compared in the same cells recorded intracellularly before and during STN HFS (Fig. 7C–F).

14 pyramidal cells were successively recorded in SNc-lesioned rats, before and during STN HFS. Before STN HFS, the mean membrane potential of the cells was −57.9±1.0 mV (*n* = 14). The STN HFS-induced decreased activity in these neurons was associated with changes in the passive membrane properties of the cells since the membrane potential was significantly hyperpolarized at −62.2±1.0 mV (*n* = 14; *p = *0.001, Paired t-test). Among those 14 recorded cells, 6 were antidromically identified as cortico-STN whereas 5 were found as projecting to the striatum. SNc lesion did not induce a difference between the membrane potentials of cortico-STN (−57.7±1.0 mV; *n* = 6) and cortico-striatal (−58.5±0.7 mV; *n* = 5) cells (*p* = 0.660, t-test). As observed for the whole cell population, STN HFS significantly hyperpolarized both the cortico-STN (−62.7±0.6 mV; *n* = 6; *p* = 0.009, Paired t-test, Fig. 7E) and the cortico-striatal cells (−63.2±0.9 mV; *n* = 5; *p* = 0.033, Paired t-test, Fig. 7F) compared to prior STN HFS (Fig. 7C–D). In addition, STN HFS had a significant effect in reducing the membrane oscillations in pyramidal cells. Indeed, in SNc-lesioned rats, the membrane potential fluctuations of cortical cells followed a Gaussian distribution which was narrowed during STN HFS. Accordingly, the value “w” calculated by the Gaussian fit was statistically reduced, indicating a sharper distribution of the membrane potential (w = 4.7±0.2 before STN HFS (*n* = 27) and w = 3.7±0.2 during HFS (*n* = 14); *p* = 0.001, t-test; Fig. 4C–E).

To reveal possible changes in the intrinsic excitability of pyramidal neurons due to HFS, we examined the V-I relationship. The input resistance was significantly decreased during STN HFS (15.7±1.7 MΩ vs 20.3±1.7 MΩ before STN HFS, *n* = 7; *p* = 0.001; paired t-test).

## Discussion

Combining *in vivo* extra- and intracellular recordings, we investigated the effects of SNc lesions on the electrophysiological properties of identified pyramidal neurons from the motor cortex. We showed that DA loss in BG resulted in a marked increase of the spontaneous discharge of pyramidal neurons as well as a profound alteration of their pattern of discharge accompanied by changes of their electrical membrane properties. Interestingly, all modifications induced by DA loss were more pronounced in cortico-striatal neurons than in cortico-STN neurons. Furthermore, STN HFS applied at parameters alleviating parkinsonian signs regularized the firing pattern of pyramidal cells and restored their electrical membrane properties.

### DA loss in BG induces an increase of the spontaneous activity and bursting pattern of pyramidal neurons in the motor cortex

According to the classical pathophysiological model of PD [1], [2], loss of DA nigro-striatal transmission is expected to increase STN and then BG output nuclei activity, ultimately leading to an over-inhibition of the thalamo-cortical network. Electrophysiological recordings performed in PD patients and animal models have confirmed the occurrence of prominent alterations in firing rate and pattern of discharge of STN cells, but the outcome of these changes in thalamo-cortical networks remains unclear. In monkey MPTP model of PD, electrophysiological recordings in thalamus and cortex failed to reveal significant changes in neuronal firing rate [8], [9], [48], [49]. In rat rendered parkinsonian by a 6-OHDA lesion, depressed spontaneous discharge activities of cortico-striatal neurons [11] and cortico-STN neurons [12] were reported using respectively single-unit and multi-unit recordings. At odds, in PD patients, functional magnetic resonance imaging (MRI) studies reported overactivity of cortical areas implicated in motor control [13], [18]. Similarly, TMS studies performed in parkinsonian patients concluded to cortical hyperactivity resulting from a decrease of intracortical inhibition originating in a default of GABAergic interneurons activation [14], [50], [51].

Here, we provide evidence in rat that a lesion restricted to the SNc resulted in a marked increase in the mean firing rate and bursting pattern of motor cortex pyramidal neurons. The discrepancy between this finding and the inhibitory effect reported by Ballion et al. [11] in urethane anesthetized rats likely arises from the experimental situations which differ both in term of extent of lesion and anesthesia. In this study, single-unit recordings were obtained under fentanyl, a powerful analgesic and sedative drug preserving a wide range of the thalamo-cortical network activities [24], [28], [52]. In addition, urethane is known to induce stereotyped slow oscillations in the membrane potential of cortical neurons consisting of up and down states transitions at 1 Hz. Such oscillations which are propagated in BG circuits are characteristic of the electrical behavior of the cortico-striatal network during slow wave sleep [53]. This anesthetic driven slow cortical rhythm might overwhelm some of the functional consequences of 6-OHDA lesion. For instance, the excessive synchronization in the beta frequency band observed in BG and cerebral cortex of both awake patients and animal models is masked in urethane anesthetized rats, except when the cortex is artificially desynchronized by applying a strong sensory input [54]. As reported in our study (Fig. 2B2 and 3B2), this cardinal sign of SNc lesion is readily observed in rats anaesthetized with fentanyl.

How to reconcile the classical PD pathophysiological model with the observed cortical hyperactivity? The cerebral cortex is a heterogeneous structure that contains both glutamatergic pyramidal (efferent) neurons and interneurons that are mostly GABAergic and play a crucial role in regulating the pyramidal cell excitability [55]. Thus, depending on which cortical neuronal population is the most efficiently influenced, the consequence of DA loss in BG might have a completely different functional impact. It is well established that axons from the thalamus make stronger and more frequent excitatory connections onto inhibitory interneurons than onto pyramidal cells [56]. It has been demonstrated that thalamo-cortical excitatory currents rose quickly in interneurons, but slowly in pyramidal cells, overlapping with feedforward inhibitory currents that suppress action potentials. Considering the rather weak increased discharge rate of BG output nuclei induced by DA loss and the limited portion of motor thalamo-cortical neurons targeted by BG output nuclei, a moderate impact on thalamo-cortical activity might primarily affect the activity of cortical interneurons. Supporting this view, Paz et al. [24] showed that following a desinhibition of thalamo-cortical neurons induced by a blockade of the glutamatergic transmission in the SNr, pyramidal neurons are not activated as would be expected by the increased activity of their thalamic afferents, but inhibited. In agreement with these results, TMS studies in PD patients concluded to a cortical hyperactivity resulting from a decrease of intracortical inhibition involving a default of GABAergic interneurons activation [14], [50], [51]. Thus, in DA loss situation, the decreased activity of thalamo-cortical neurons predicted by the PD model is compatible with our observation of a hyper-excitability of pyramidal neurons. The overactivity of pyramidal cells is likely to participate in the hyperactivity of glutamatergic cortico-striatal transmission reported in PD states [57]–[61].

In addition to increase the firing rate of cortical pyramidal cells, DA loss in BG induced profound alterations in their discharge pattern, as assessed by Poisson Surprise analysis. The cell discharges became more irregular with an increased mean bursts occurrence and a higher mean intraburst frequency. These alterations cannot be related to a change of the cell type since the proportions of RS, NIB and IB cells were not modified following the SNc lesion. These changes might stem from the propagation of altered pattern of neuronal discharge in BG output nuclei through the thalamus. Indeed, numerous studies have reported a dramatic change of BG output structures activity in PD patients as well as in animal models [62]–[64] including a more irregular discharge with bursts and pauses as well as increased oscillations and synchronicity. Alterations of discharge observed in pyramidal neurons, which also involved an increased irregularity, were similar to the alterations recorded in BG output structures.

Noteworthy, DA loss impacted more cortico-striatal neurons than cortico-STN ones, both in terms of spontaneous activity and electrical membrane properties. This phenomenon remains unexplained but several hypotheses might account for these differences. First, the two cell populations could express distinct voltage gated channels underlying distinct electrophysiological properties. However, no such distinct electrophysiological profiles have been yet demonstrated [30]. Alternatively, a second hypothesis might rely on the differential cortical location of cortico-striatal and cortico-STN neurons. Cortico-STN neurons lay in deep layers whereas cortico-striatal cells are in more superficial layers [30], [31], [65]. Anatomically, it is well established that BG thalamic recipient nuclei, such as the ventral medial nucleus, which receive inputs from the SNr, innervate the superficial cortical layers [66] and therefore might have a more prominent action on neurons lying in superficial layers.

### STN HFS reversed alterations induced by DA loss

STN HFS, a deep brain stimulation (DBS) procedure, was able to counteract the changes in discharge pattern, firing rate and intrinsic properties of pyramidal cells induced by DA lesion. The effect on the firing pattern, i.e. a decrease of bursts, is reminiscent of the regularization of the pattern of discharge of BG output nuclei during STN HFS observed in PD patients and experimental models [6], [7], [67], [68]. Such regularization has been considered as a major mechanism by which STN HFS alleviates parkinsonian motor symptoms. By reducing the noise generated in the motor thalamus, STN HFS would allow the encoding of coherent motor commands by motor cortex. The present observation at cortical level provides an additional support to this view.

Our data documented that STN HFS induces an antidromic activation of pyramidal neurons of the motor cortex in agreement with previous studies [12], [46], [69]. The central role of the antidromic activation of the cortex in the beneficial effects of STN HFS has been well documented in other recent studies [12], [69], [70]. As a consequence, antidromic spikes at 125–130 Hz propagate in the local collateral network of cortico-STN neurons which might target pyramidal cells as well as GABAergic interneurons [12]. In addition, STN stimulation might also activate the recently described STN-cortex pathway which innervates the motor area [34]. Since inputs could activate inhibitory interneurons more strongly than pyramidal cells, triggering powerful feedforward inhibition, it is expected that STN HFS activate preferentially interneurons resulting in a powerful decrease of cortical excitability, thus counteracting deleterious effects of DA loss. Such a mechanism is favored by the fact that the reduction of pyramidal cell firing is accompanied by a decrease in membrane resistance, an effect consistent with a GABAergic inhibition. This seems at odds with a recent study which, by combining DBS and TMS, reported a cortical activation following STN stimulation [71]. However, this study tested STN stimulations at low frequencies (3 and 30 Hz) whereas frequencies >100 Hz are necessary for effective STN DBS. Our results are consistent with other TMS studies showing that the default of GABAergic interneurons activation observed in PD patients is restored by STN HFS [14], [51], [72], [73]. Further studies are required to directly verify this hypothesis.
